# MicroRNA-regulated B cells in obesity

**DOI:** 10.1097/IN9.0000000000000005

**Published:** 2022-08-05

**Authors:** Alyssa J. Matz, Lili Qu, Keaton Karlinsey, Beiyan Zhou

**Affiliations:** 1 Department of Immunology, School of Medicine, University of Connecticut, Farmington, CT, USA; 2 Institute for Systems Genomics, University of Connecticut, Farmington, CT, USA

**Keywords:** B cell, microRNA, adipose tissue, obesity

## Abstract

Obesity is a prevalent health risk by inducing chronic, low-grade inflammation and insulin resistance, in part from adipose tissue inflammation perpetuated by activated B cells and other resident immune cells. However, regulatory mechanisms controlling B-cell actions in adipose tissue remain poorly understood, limiting therapeutic innovations. MicroRNAs are potent regulators of immune cell dynamics through fine-tuning a network of downstream genes in multiple signaling pathways. In particular, miR-150 is crucial to B-cell development and suppresses obesity-associated inflammation via regulating adipose tissue B-cell function. Herein, we review the effect of microRNAs on B-cell development, activation, and function and highlight miR-150-regulated B-cell actions during obesity which modulate systemic inflammation and insulin resistance. In this way, we hope to promote translational discoveries that mitigate obesity-induced health risks by targeting microRNA-regulated B-cell actions.

## 1. Introduction

Obesity is a risk factor for an expanding set of chronic comorbidities, increasing global rates of disability, and mortality ^[1–3]^. White visceral adipose tissue (VAT) dysfunction plays a central role, bolstering local and systemic inflammation and insulin resistance ^[4]^. Studies demonstrate anti-inflammatory treatments in obese individuals decrease the risk of cardiovascular disease and other comorbidities ^[5–8]^; however, targeted approaches to decrease inflammation are necessary to limit deleterious effects.

During obesity, the VATs immune cell compartment expands, with B cells becoming the second most abundant immune lineage behind macrophages ^[9–15]^. B-cell depletion ameliorates obesity-associated metabolic dysfunction; however, limited understanding of molecular mechanisms regulating B-cell actions under obesity stress has hampered the translational potential of targeting B-cell–mediated inflammation to reduce obesity-associated disease risk. The epigenetic factor miR-150 presents a potent regulator of B-cell differentiation, activation, and function ^[12,16–18]^; its expression can modulate obesity-induced inflammation and insulin resistance in a B-cell–dependent manner ^[12]^. Thus, in this review, we detail current knowledge on B-cell actions regulated by microRNAs and integrate the impact of microRNA-regulated B cells actions in obesity, cancer, and autoimmunity. In this way, we demonstrate the potential of microRNAs as specific, targetable mediators of obesity-induced health risk, with an emphasis on miR-150 in adipose tissue B cells.

## 2. MicroRNAs are crucial regulators of B cell biology and B-cell–mediated diseases

### 2. 1. General introduction to microRNAs

MicroRNAs are small, non-coding RNAs that provide important epigenetic regulation of cell development, activation, and function ^[19]^. Functional microRNAs are part of a cytoplasmic RNA-induced silencing complex (RISC), where the single-stranded microRNA binds complementary mRNA targets and inhibits translation ^[20]^. For a comprehensive review of microRNA biogenesis, see Mehta and Baltimore ^[19]^ and Ha and Kim ^[21]^. A note on nomenclature, pre-microRNAs are encoded as hair-pin loop structured RNAs that are processed to select for one functional strand (mature microRNAs), denoted as the 5p or 3p strand ^[22]^. Typically, one of the strands is dominant in a cell lineage. For miR-150, the -5p strand is dominant compared to its -3p complement ^[23]^. In addition, microRNAs of distinct sequences can be encoded in the genome as clusters under the same promoters as a polycistronic transcript, like the miR-17~92 cluster ^[24]^. Furthermore, identical microRNA sequences can be transcribed in different locations in the genome and are denoted by the suffix -1/2/3/, etc (eg, miR-let-7a-1 and miR-let-7a-2), while microRNAs with slightly different sequences or lengths, known as isomiRs, are denoted with the same number but with the suffix of a/b/c/, etc (eg, miR-let-7a and miR-let-7d) ^[25]^.

MicroRNA targeting specificity is dictated by their first 7–8 nucleotides on the 5′ end of the microRNA, known as the “seed” nucleotides ^[26]^. Every microRNA targets numerous mRNAs and over 60% of all proteins are predicted to be under microRNA regulation ^[27,28]^. Several algorithms are available to predict microRNA-regulated genes including TargetScan ^[29]^, miRDB ^[30]^, PicTar ^[31]^, RNA22 ^[28]^, and microT-CDS ^[32]^, and others that adopt various algorithms with different weights of miRNA-target interaction factors. Consideration to the level of complementary of the target site within the mRNA is central to predicting the efficiency of microRNA-mediated mRNA repression ^[33]^. In addition, the region targeted is important, with sites in the 3′ untranslated region (UTR) yielding the greatest repression ^[33]^. Furthermore, microRNAs are highly conserved across species, thus target regions with conservation are considered more relevant for microRNA-mediated repression ^[33]^. However, microRNA-mediated mRNA repression should be validated in all species of interest; thus, in this review, the species a study utilized is indicated using typical naming conventions. Predicted targets can also be cell-specific as both microRNA and targets must be expressed to be biologically relevant.

These considerations underpin the importance of validating predicted targets in the cell and species of interest through functional analyses. First, microRNA and target expression patterns can be determined to establish a relationship. Ideally, the microRNA-mediated repression of translation is validated; many scientists make use of luciferase reporter constructs containing the mRNAs targeted region and transfected microRNA. Additionally, the targeted regions can be mutated to limit microRNA repression to observe the effect of microRNA regulation of a single target on a process of interest.

### 2. 2. MicroRNAs important to B cells

Since the discovery of microRNAs in regulating hematopoiesis and immune cell function, a set of microRNAs have been identified as key regulators for B-cell formation and function. Herein, we detail important microRNA networks across B-cell stages and actions (Table 1). The potency of these networks is exemplified in disease contexts, as genetic- or epigenetic-altered microRNA expression can drive B-cell malignancies, autoimmunity, and sub-optimal immune responses. One of the earliest studies of microRNAs in B cells investigated the deletion at chromosome 13q14 that occurs in over half of all B-cell chronic lymphocytic leukemia (B-CLL), mantle cell lymphoma, and other non–B-cell cancers ^[34,35]^. MiR-15 and miR-16 are encoded in this large region and both genes are deleted or down-regulated in the majority of CLL cases ^[35]^. Both miR-15a and miR-16-1 target apoptosis regulator Bcl2 (*BCL2*) to promote cell death, acting as tumor suppressors ^[36]^. This discovery demonstrates a causal role in altered expression of this and other microRNAs ^[36,37]^ for cell transformation. Following this, microRNA expression patterns have been leveraged as prognostic factors for survival outcomes or response to treatment ^[35,38–40]^.

**Table 1:** Select microRNAs networks important to B-cell function and appreciated in B-cell–mediated diseases.

microRNA	Impact on B cells	Validated Targets	Disease contexts	References
miR-150	Pro- to pre-B-cell transition	*MYB*	Obesity, cancer, autoimmunity	^[12,16–18,41–44]^
	Negative regulator of BCR signaling	*MYB, FOXP1, GAB1, Etf1, Elk1*		
miR-15	Negative regulator of B-cell proliferation; important for Germinal center response	*Bcl2*	Cancer	^[35,36]^
miR-16				
miR-34a	Pro- to pre-B-cell transition	*FOXP1*	Cancer	^[45]^
miR-146a	Negative regulator of B-cell proliferation; important for central tolerance	*Fas*	Autoimmunity	^[46,47]^
miR-148a	Negative regulator of B-cell apoptosis; important for central tolerance	*PTEN, Gadd45α, Bim*	Autoimmunity	^[39]^
miR-17	Negative regulator of pro-inflammatory signaling in Leukemic B cells	*TNFA, TLR7*	Cancer	^[48,49]^
miR-19a				
miR-155	Negative regulator of affinity maturation and isotype switch	*AID, PU.1, SHIP-1, HGAL, C/EBPb, SMAD5*	Cancer, autoimmunity	^[50–58]^

BCR, B-cell receptor.

### 2.3 MicroRNAs in hematopoiesis and B-cell development

Adult B cells predominantly arise from hematopoietic stem cells (HSCs) within the bone marrow, known as B2 or follicular B cells ^[59]^. In addition, mice have an identifiable self-replenishing, rapid-responding B cells subpopulation, termed B1 cells, derived from the fetal liver; mouse B1 cells are enriched in body cavities and contribute to rapid, poly-specific antibodies, reviewed elsewhere ^[59]^. While functional human counterparts exist, the origins of these B-cell populations in humans and other species remain less defined ^[55]^. Differential expression of the microRNA let-7 family contributes to the differential affinity of fetal and hematopoietic progenitor cells to yield B2 versus B1 phenotypes ^[60,61]^.

Hematopoiesis is a continuum of maturation regulated by a network of transcription factors. Transcription factor levels are tightly regulated to initiate and intensify lineage commitment. MicroRNAs can establish a threshold for transcription factor mRNA expression necessary to drive a cell down a particular lineage differentiation cascade ^[19]^. HSCs within bone marrow differentiate into several lineages of blood cells, including immature B cells. From the common lymphoid progenitor stage into B-cell lineage commitment, cells first rearrange and assemble a productive immunoglobulin heavy chain, termed the pro-B phase ^[62,63]^. Signaling of successful heavy chain assembly initiates proliferation (known as the large pre-B phase) and subsequent recombination of the light chain (small pre-B) ^[64]^. Light chains are paired with rearranged heavy chains to form the B-cell receptor (BCR) ^[65,66]^. Small pre-B cells display BCRs on the cell surface to interact with self-antigens and, provided the signal strength, will either (a) undergo positive selection, (b) receive insufficient signal and continue light chain rearrangement, (c) ligate self-antigens forcing receptor editing or apoptosis ^[67]^. For further review of B-cell hematopoiesis, see Hardy and Hayakawa ^[67]^.

The first study to implicate microRNAs in hematopoiesis identified that miR-181a, miR-142, and miR-223 are differentially expressed in hematopoietic tissue and across lineages ^[68]^. Expression of miR-181a is highest in the thymus and the B-cell lineage of the bone marrow. Overexpression of miR-181a in HSCs increased the proportion of B cells generated ^[68]^. Later studies demonstrated miR-181a expression is necessary for development to the pro-B stage of B-cell commitment and decreases in subsequent B-cell lineage stages while increasing in T cells ^[16]^. In opposition, miR-150 increases starting at the pro-B stage, with the highest expression in mature naive B cells ^[16]^. MiR-150 overexpression in HSCs decreases the proportion of circulating B cells ^[16]^, while miR-150 knock-out likewise reduced the proportion of B2 cells compared to B1s ^[41]^. Although miR-150 overexpression generates regular pro-B numbers, subsequent pre-B and later stages exhibit increased rates of apoptosis ^[16]^.

**Figure 1.**
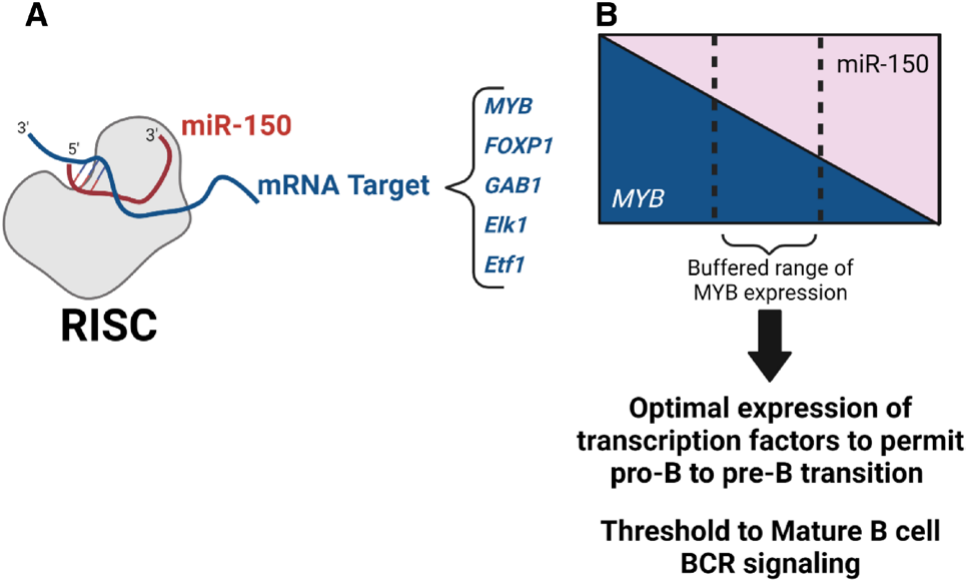
MiR-150 targets B-cell transcription to permit B-cell development and establish a threshold to mature B-cell BCR signaling. (A) Representation of mature miR-150 within RISC targeting mRNA targets relevant to B cells including *MYB, FOXP1, GAB1, Elk1,* and *Etf11*. (B) Representation of miR-150 buffering MYB protein expression to achieve an optimal range to regulate functions. Created with BioRender.com. BCR, B-cell receptor; RISC, RNA-induced silencing complex.

MiR-150 has several targets validated by similar workflows in both humans and rodents. MiR-150 repression of Transcriptional activator Myb (*MYB*) ^[16,41]^ is most relevant in B-cell differentiation. In addition, *MYB*, transcription factor forkhead box protein P1 (*FOXP1*) ^[17,18]^, GRB2-associated-binding protein 1 (*GAB1*) ^[18]^, ETS domain-containing protein Elk-1 (*Elk1*) ^[12]^, and Eukaryotic peptide chain release factor subunit 1 (*Etf1*) ^[12]^ are part of the miR-150 network that regulates mature B-cell actions (Figure 1A). MiR-150 is highly specific to hematopoietic cells ^[23,69]^. In addition to B-cells, miR-150 regulation is important to megakaryocyte-erythrocyte progenitor differentiation ^[70]^, Natural Killer cell generation and function ^[71,72]^, and T-cell development ^[16,73]^. Additional miR-150 networks in immune cells include miR-150 repression of E3 ubiquitin-protein ligase CBL (CBL) and E3 SUMO-protein ligase EGR2 (EGR2) mRNAs-induced apoptosis in mixed-lineage leukemia cells ^[74]^ and repression of C-C chemokine receptor type 6 (CCR6) to limit cutaneous T-cell lymphoma invasion/metastasis ^[75]^.

As stated, miR-150 targets *MYB* and *FOXP1*, essential transcription factors in human and mouse B cells ^[18,41]^. MiR-150 expression is inversely correlated with *MYB* in B-cell development and *MYB* deletion leads to a severe block of B-cell development at the pro- to pre-B transition ^[76]^, suggesting miR-150 regulation of *MYB* is important for B-cell development. MiR-150 is considered to buffer *MYB* expression to a small range for sufficient *MYB* protein expression for B-cell development while preventing excessive expression leading to dysregulated expansion and leukemia ^[19]^ (Figure 1B). In opposition, *Foxp1* expression is higher in pre-B compared to pro-B in mice; however, goes down in mature B cells ^[45]^, suggesting miR-150 regulation of *Foxp1* may be more relevant in mature B cells. MiR-34a also targets *Foxp1* in mice and decreases in pre-B, and constitutive expression decreases pre-B-cell numbers ^[45]^. MiR-150 and miR-34a may cooperate to repress *Foxp1* and present an interesting network of B-cell development regulation. Additional microRNAs have been implicated at all stages of hematopoiesis including myeloid versus lymphoid divergence and B-cell versus T-cell lineage commitment; for review of microRNA regulation of hematopoiesis, see Havelange and Garzon ^[77]^.

### 2. 4. MicroRNA-regulated B-cell tolerance and autoimmune disease

B cells displaying auto-reactive BCRs are deleted or inhibited through central and peripheral tolerance. Elimination of auto-reactive B cells in the bone marrow is termed central tolerance ^[78]^. Upon leaving the bone marrow niche, immature B cells undergo peripheral tolerance to inhibit auto-reactive B cells and mature into functional B cells. Peripheral tolerance can result from similar exposure to auto-antigens; however, B cells may undergo anergy, antigen receptor desensitization, or tolerance to antigen through engagement of inhibitory receptors. Escape from tolerance mechanisms is implicated in mature B-cell malignancies and autoimmune and rheumatological diseases such as systemic lupus erythematosus (SLE) and rheumatoid arthritis. For further review of B-cell tolerance, see Nemazee ^[78]^.

In particular, miR-146a is a major negative regulator of inflammatory responses in immune cells and is significantly down-regulated in SLE ^[79]^. The importance of miR-146a to the immune response was first demonstrated in myeloid cells, where miR-146a imposes tolerance to pro-inflammatory cytokines and danger-associated molecular patterns by targeting key cytokine-receptor and TLR adaptor molecules interleukin 1 receptor-associated kinase 1 (*IRAK1*) and TNF receptor-associated factor 6 (*TRAF6*) ^[80]^. In B cells, miR-146a was found to target tumor necrosis factor receptor superfamily member 6 (*Fas*) ^[46]^. Overexpression of miR-146a limits B-cell apoptosis, promoting symptoms of the autoimmune lymphoproliferative syndrome ^[46]^. Furthermore, the SLE risk allele rs2431697 resides in the regulatory element for miR-146a and impairs miR-146a expression in a cell-lineage-dependent manner ^[79]^.

Phosphatidylinositol 3,4,5-trisphosphate 3-phosphatase and dual-specificity protein phosphatase (PTEN) is another critical signaling molecule in B cells that is hypo-responsive in SLE B cells ^[81]^. MiR-148a targets *PTEN* and is commonly upregulated in patients with SLE and Lupus-prone mice ^[39]^. miR-148a also targets Growth arrest and DNA damage-inducible protein GADD45 alpha (*Gadd45α*), and Bcl-2-like protein 11 (*Bim*), blocking B-cell apoptosis and allowing auto-reactive B cells to escape central tolerance ^[39]^. MiR-150 has been suggested to establish a threshold for BCR signaling ^[18]^, an important part of central tolerance. Further, miR-150 expression patterns have been linked to myasthenia gravis ^[42,43]^ and autoimmune hemolytic anemia ^[44]^.

### 2. 5. MicroRNA-regulated mature B-cell functions

B cells play central roles in tissue homeostasis in response to exogenous and endogenous stimuli. Mature B cells present antigen to CD4^+^ T cells, contribute to lymphoid tissue homeostasis and immune response regulation through cytokine production, and are solely responsible for antibody secretion ^[82]^. Their actions under chronic or acute conditions can be drastically different concerning the speed of initiation, signaling intensity, and immune outputs. For example, B-cell dysfunction can develop under chronic conditions such as elderly, obese, or in immunocompromised individuals, leading to impaired biodefense and severe infections ^[82,83]^. Alternatively, B cells can be overactive, contributing to asthmatic and autoimmune conditions ^[82]^. MicroRNA expression is crucial to fine-tune these responses and permit productive immune activation.

Upon BCR ligation, mature B cells upregulate antigen processing and presentation on major histocompatibility complex II (MHCII) ^[84]^. Antigen-presenting B cells provide MHCII + antigen and co-stimulatory signals to cognate CD4^+^ T cells to permit both B-cell development and T-cell activation. Few studies have investigated microRNA regulation in the antigen presentation network, for example ^[85,86]^, and none have used B-cell-antigen presentation.

B-cell cytokine production is vital for immunomodulatory effects of B cells within tissues during an immune response ^[87]^. Functional B-cell subsets are defined by major cytokines produced, including regulatory B cells (B_regs_) which maintain high expression of anti-inflammatory interleukin-10 (IL-10) ^[87]^. Effector B cells are denoted by expression of pro-inflammatory cytokines that promote T-cell and macrophage activation, including tumor necrosis factor-alpha (TNFα) ^[87]^. The microRNA polycistronic miR-17~92 cluster is often amplified and overexpressed in diffuse large B-cell lymphomas and other cancers ^[48]^. In B-CLL cells, miR-17 and miR-19a of the miR-17~92 cluster can both independently target *TNFA* and toll-like receptor 7 (*TLR7*), reducing TLR7-induced proliferation ^[49]^. The importance of this cluster in normal B-cell cytokine production has yet to be established.

Following activation, B cells can undergo differentiation to plasma cells to secrete abundant antibodies. To generate highly antigen-specific antibodies, B cells must first differentiate into germinal center B cells (GCBs). Following B cell–T cell interactions, in which B cells receive additional activating signaling (CD40 ligation and IL-4/IL-21 cytokines), B cells may differentiate into GCBs where they undergo proliferation, class-switch recombination (CSR), somatic hypermutation (SHM), and clonal selection. These actions of GCBs are coordinated by a network of transcription factors under the regulation of multiple microRNAs including miR-155, miR-181b ^[88]^, miR-30 ^[89]^, miR-9 ^[89]^, let-7 family members ^[39,89]^, and miR-125b ^[90]^. MiR-150 expression decreases in GCBs compared to naive and memory B cells, inversely correlating with the expression of *MYB* ^[91]^. Functional implications of this trend have not been explored.

Of note, miR-155 is a common immune regulator across myeloid and lymphoid cells ^[92]^ and deficiency leads to fewer extra follicular germinal centers and reduced high-affinity antibody selection ^[50]^. MiR-155 has several validated targets in B cells important to GCB reactions including activation-induced cytidine deaminase (AID) ^[51,52]^, a potent DNA mutation crucial to both CSR and SHM ^[93]^, and transcription factor PU.1 (*SPI1*) ^[53]^, an important regulator of plasma cell differentiation through control of paired box protein Pax-5 (*PAX5*). MiR-155 regulation has been explored across many diseases in various immune cells; for further information, see Vigorito et al ^[94]^.

### 2. 6. MicroRNA-regulated adipose tissue B-cell function in obesity

The B cell population in adipose tissue expands during obesity in both mice and humans ^[15]^. However, knowledge of their functions and regulatory mechanisms are less defined. The Engleman lab in 2011 was the first to implicate B cell actions in driving obesity-associated health risks ^[10]^. In models of diet-induced obesity (DIO), B-cell deficient mice (either through germline mutation or antibody-mediated depletion) have improved measures of glucose tolerance and insulin resistance ^[10]^. B-cell deficiency limited pro-inflammatory macrophage expansion in obese VAT, likely through cytokine actions, and was dependent on B-cell ability to present antigen to T cells ^[10]^. Furthermore, a unique antibody profile is observed in obese plasma that independently worsened metabolic health upon transfer to obese B cell deficient mice ^[10]^. However, the contributions of varying B-cell actions and immunoglobulin classes in obesity are needed; indeed, IgA-producing B cells in the intestines are important for barrier maintenance and can impact glucose homeostasis ^[13]^. Furthermore, anti-lipid immunoglobulins are an important mechanism for clearing atherogenic oxidized lipids ^[95]^. Interestingly, auto-reactive anti-insulin immunoglobulins are present in humans despite normal glucose levels and actually contribute to glucose homeostasis by both eliminating and protecting insulin depending on the immunoglobulin class ^[96]^. Thus, greater understanding into crucial regulatory mechanisms driving obesity-associated B-cell cytokine production and unique antibody profile is needed to innovate targeted therapeutic approaches.

To explore regulatory mechanisms of B cell actions in obesity, our laboratory utilized miR-150 knock-out (miR-150KO) mice ^[41]^, which have normal physiology and altered plasma antibody profiles ^[12]^. During DIO, miR-150KO mice exhibit more severe inflammation and glucose intolerance ^[12]^. Adoptive transfer of lean miR-150KO B cells to WT obese mice exacerbated metabolic dysfunction, demonstrating that miR-150 regulated B-cell actions are important in this context ^[12]^. Transfer of miR-150KO plasma antibodies was not sufficient to replicate worsened metabolic parameters; however, miR-150KO B cells were found to undergo increased T-dependent activation and induce increased activation of macrophages and T cells in co-culture experiments ^[12]^. Together, these results implicate miR-150 regulated B-cell immune modulation in obesity-induced health risk. We further implicated miR-150 in BCR signaling, revealing increased BCR signaling components present at a steady state ^[12]^. This increase was mediated through miR-150 targeting *Myb*, *Elk1*, and *Etf1* ^[12]^. Additionally, miR-150 regulation of *FOXP1* and *GAB1* has been demonstrated to limit BCR signaling ^[18]^. The importance of miR-150s impact on BCR signaling in adipose tissue B cells to obesity-induced health risk has yet to be established. Thus, miR-150 is a potent regulator of B-cell development and activation, and miR-150-regulated B-cell actions are important for obesity-induced systemic inflammation and insulin resistance. However, key questions remain about the regulatory mechanisms of miR-150 in adipose tissue B cells during obesity and subsequent obesity-induced health risk.

## 3. Perspective

The global incidence of obesity comorbidity and mortality continues to rise, necessitating targeted therapeutic strategies to limit obesity-induced inflammation and insulin resistance. B cells are important drivers of obesity-induced health risk; however, the heterogeneous actions of B cells require targeted approaches to translate this knowledge into therapy. Thus, the molecular mechanisms underpinning B-cell actions driving obesity-associated disease risk should be investigated. MicroRNAs are potent regulators of the immune response and provide a potential target to alter specific cell actions. In particular, miR-150 is a crucial regulator of B cells and miR-150 regulation is important to B-cell actions in obesity. Nuanced microRNA regulation of adipose tissue B cells and other immune cells during obesity, particularly by miR-150, demands exploration to both uncover important networks and generate novel therapeutic targets.

## Author contributions

AM and BZ wrote and revised the manuscript. LQ and KK contributed to manuscript design and revisions. All authors have read and agreed to the published version of the manuscript.

## Conflict of interest

The authors declare that they have no conflicts of interest.

## Funding

This research was funded by the American Heart Association, grant number 19TPA34910079, and National Institutes of Health/National Institute of Diabetes and Digestive and Kidney Diseases, grant number R01DK121805 to BZ.
